# Patient-reported outcomes from a workplace intervention program for cancer survivors highlight ongoing needs to support continuation of work

**DOI:** 10.1007/s00520-019-04964-1

**Published:** 2019-07-08

**Authors:** Dawn Aubel

**Affiliations:** Novartis Oncology, 161 South Finley Ave, Basking Ridge, East Hanover, NJ USA

**Keywords:** Patient-reported outcomes, Self-efficacy, Social support, Patient navigation, Nurses, Cancer survivors

## Abstract

**Purpose:**

The aim of this study was to investigate the perceptions of cancer survivors who continue to work and provide information to evaluate and develop a supportive workplace program (Ensemble) based on the principles of navigation.

**Methods:**

A mixed-methods design using surveys and open-ended questions was used to study the perceptions of two groups of cancer survivors in the same workplace: those who chose to use a workplace navigational program (Ensemble program users) and those who declined (non-users). Key outcomes were communication and attitudinal self-efficacy, measured by the Communication and Attitudinal Self-Efficacy scale for cancer (CASE-cancer); emotional and informational social support, measured by the Patient-Reported Outcomes Measurement Information System Social Support domain (PROMIS-Social Support); and satisfaction with the navigator relationship, measured using the Patient Satisfaction with Interpersonal Relationship with Navigator (PSN-I).

**Results:**

The study included 7 program users and 17 non-users. There were no significant differences in attitudinal self-efficacy, emotional support, or informational support between the groups. The relationship with the Nurse Navigator was rated highly by program users. The most frequent themes to the open-ended responses included work demands, privacy, integration of life and work, and program improvement.

**Conclusions:**

Successful reintegration into/continuation of work remains a key need for cancer survivors. The navigation program design for cancer survivors should be further improved and applied across work settings.

**Electronic supplementary material:**

The online version of this article (10.1007/s00520-019-04964-1) contains supplementary material, which is available to authorized users.

## Introduction

Advances in cancer diagnosis and treatment have resulted in declining cancer mortality rates during the past 20 years [1], resulting in high rates of return to work following a cancer diagnosis [2]. Cancer survivors report that work provides a sense of normalcy, necessary financial support, and in some cases, social support [3]. Cancer survivors who work after diagnosis have unique needs to ensure optimal outcomes for both the individual and the employer. A supportive work environment is an important factor for success [4]. Work-related factors significantly associated with a greater likelihood of employment or return to work after cancer diagnosis include perceived employer accommodation, flexible work arrangements, and supportive services such as counseling, training, and rehabilitation [4].

There is a lack of conclusive evidence from peer-reviewed studies on effective, sustainable solutions that may be implemented in the workplace for cancer survivors [5]. One way that employers can help employees manage cancer is by providing support in the form of navigation. In general, patient navigators assist patients in overcoming challenges in the healthcare system and can also assist with the challenges of cancer survivorship [6]. A recent systematic review of patient navigator programs for chronic disease found significant heterogeneity in outcomes among the 67 studies evaluated, 44 of which were on cancer [7]. Although no studies found a negative impact, the variability of the literature precludes conclusions regarding the effectiveness of patient navigation programs [7].

We used Ensemble, a workplace patient navigation program that utilizes a Nurse Navigator, to investigate the perceptions of cancer survivors in the workplace. The Ensemble program provides emotional and informational support, along with health coaching, to help participants cope with the complex demands of balancing work and a cancer diagnosis. The study assessed two groups of cancer survivors in the workplace: those who participated in Ensemble and those who declined to participate. The information and insight gained from this study were used to develop a supportive workplace program based on the principles of navigation.

## Methods

### Study design

A mixed-methods design using surveys and open-ended questions was used to study the perceptions of two groups of cancer survivors in the same workplace: those who chose to use a workplace navigational program (Ensemble) and those who declined to use the program (Online Resource 1). Key outcomes were communication and attitudinal self-efficacy, measured by the Communication and Attitudinal Self-Efficacy scale for cancer (CASE-cancer); emotional and informational social support, measured by the Patient-Reported Outcomes Measurement Information System Social Support domain (PROMIS-Social Support); and satisfaction with the navigator relationship, measured using the Patient Satisfaction with Interpersonal Relationship with Navigator (PSN-I) [8–12].

The CASE-cancer is a 12-item questionnaire in which participants respond according to a Likert-type scale consisting of four points: 1 = strongly disagree, 2 = slightly disagree, 3 = agree, and 4 = strongly agree [8]. Total scores range from 12 to 48, with higher scores indicating higher levels of self-efficacy [8]. Self-efficacy is defined as the perceived self-management, knowledge, and confidence in processing information, making decisions, and obtaining care [8].

The PROMIS-Social Support consists of six subdomains that include 4-, 6-, or 8-item questionnaires [10]. The 8-item questionnaires from the Emotional Support and Informational Support subdomains were used in this study. Participants chose responses from a Likert-type scale consisting of five points: 1 = never, 2 = rarely, 3 = sometimes, 4 = usually, and 5 = always. Each subscale was scored separately, with a total score range of 8 to 40, with higher scores indicating higher levels of social support. Social support is defined as the feeling of being cared for and valued as a person and the availability of assistance and social ties.

The PSN-I was administered only to Ensemble program users to assess their perceptions of the navigator’s relational quality [12]. It consists of 9 items that address the adequacy of time spent with the patient; the patient’s comfort level; the navigator’s perceived dependability, courtesy, respect, and listening ability; ease of communication; perception of a caring relationship; navigator problem solving; and navigator accessibility [12].

Participants also filled out a demographic survey (Online Resource 2) and open-ended questions (Online Resource 3). Open-ended questions were designed to gather qualitative data on the perceived needs of cancer survivors who continue to work and the reasons for deciding whether or not to participate in the workplace navigational program.

### Standard protocol approvals, registrations, and patient consents

Ethical approval was obtained by the Institutional Review Boards of Teacher’s College, Columbia University, and by the legal counsel of the company of the study setting. The participants were informed of the study’s purpose and their voluntary participation. Consent was presented to participants after they met the inclusion criteria, and participants provided consent with an electronic signature. Informed consent was obtained from all individual participants included in the study.

### Navigational program

 Ensemble is a multi-faceted innovative workplace program based on a navigation model using nurses and is designed to provide guidance and support to employees who are cancer survivors, caregivers, or managers of employees affected by cancer. Ensemble aims to provide emotional and informational support along with health coaching to improve self-efficacy and better equip participants to cope with the complex demands of balancing work and a cancer diagnosis. The study reported here focused exclusively on employees who are cancer survivors. A dedicated Ensemble website was available to employees through the company’s intranet site and included internal links to pertinent company human resources and benefits policies, information detailing how to connect to Ensemble resources, and quick links to external cancer-related resources such as supportive patient advocacy groups.

The central feature of the program was the Nurse Navigator, who provided individualized care to employees who opted into the program. The Nurse Navigators were registered nurses with expertise in oncology nursing, case management, and work and cancer, and also specially trained in the organization’s policies, benefits, internal resources, and volunteer employee networks. They were available to the employees and their beneficiaries during regular business hours. For each Ensemble participant, the approximate time spent with the Nurse Navigator included a 10-min contact call, a 45- to 60-min initial assessment, subsequent encounters based on the need (consisting of emails and phone calls), and a 15-min follow-up evaluation 1 week after the final encounter. The average total time spent per Ensemble participant was 91 min, with an average of 19 encounters per Ensemble participant. To comply with privacy standards, the nursing provision, consisting of a nurse project lead and two Nurse Navigators, was contracted through an external agency, which managed all participant communications, interactive software, and medical records confidentially, securely, and separately from the company.

The program also leveraged internal medical experts such as physicians, nurses, or pharmacists as volunteers to help inform the program participants regarding cancer treatment guidelines, second opinions, and clinical trials. Volunteers completed formal training and signed a volunteer agreement form to participate, but they were not permitted to give specific advice or referrals for care. The Nurse Navigator maintained a list of volunteers with their respective areas of expertise and was responsible for referring participants who requested detailed cancer-related medical information to the appropriate volunteer(s).

### Recruitment

Study participants were cancer survivors who worked in the company where the workplace navigational program, Ensemble, was administered. To participate, individuals had to be 18 years of age or older, have a cancer diagnosis at any time of their life, and have an ability to understand and read English. Two populations were recruited: Ensemble program users and non-users. Eligible program users were invited to participate in the study by email invitation sent through the secure Ensemble portal. Non-users were invited through established company communications, including online newsletters and group email lists. Additional details related to participant flow are included in Online Resource 4.

### Data collection and analyses

The information technology vendor, Clinical Trial Media, Hauppauge, New York, programmed the software system, managed the automated survey administration, and maintained the data collected. Data were held securely and separately from the investigator and company. The information technology vendor provided raw anonymous data to the investigator. Statistical analyses were performed using the 2015 version of Statistical Package for the Social Sciences, IBM® SPSS® Statistics 23 (IBM, Armonk, New York). Independent sample *t* tests were reported for CASE-cancer and for PROMIS-Social Support’s Emotional Support and Informational Support domains. The responses to open-ended questions were entered into the qualitative software analysis program NVivo (Version 11; QSR International Pty Ltd, Doncaster, Victoria, Australia), and a word frequency query was conducted to highlight meaningful patterns. Categorizations of qualitative data were aligned to The Cancer Survivorship and Work Model components, which include work environment, individual and interpersonal, cancer and treatment, and intervention program factors [13]. (See Online Resource 5.) Words used by study participants were counted, and a word cloud was generated giving prominence to the words that appeared more frequently.

## Results

### Participants

The study included 7 participants in the Ensemble program user group and 17 in the non-user group. Demographic, work, and cancer background information is summarized in Table 1. The office setting was the most common job location reported for both program users (85.7%) and non-users (82.4%), and most participants reported work demand as demanding or very demanding (Table 1). Breast cancer was the most common primary cancer site reported for both program users (57.1%) and non-users (35.1%) (Table 1). Both groups reported a wide range of time since diagnosis and mostly > 1 treatment type (Table 1).

**Table 1:** Demographics and background characteristics

	Ensemble program users (*N* = 7)		Ensemble program non-users (*N* = 17)	
	*n*	%	*n*	%
Age, years				
35–39	1	14.3	1	5.9
40–44	0	0.0	2	11.8
45–49	1	14.3	4	23.5
50–54	3	42.8	4	23.5
55–59	2	28.6	3	17.6
60–64	0	0.0	2	11.8
Do not wish to answer	0	0.0	1	5.9
Female	4	57.2	13	76.5
Caucasian	7	100.0	15	88.2
Marital status				
Single/never married	2	28.6	2	11.8
Married/living as married	3	42.8	12	70.5
Divorced/separated	2	28.6	1	5.9
Widowed	0	0.0	1	5.9
Do not wish to answer	0	0.0	1	5.9
Level of education				
Associate’s degree	1	14.3	2	11.8
Baccalaureate degree	4	57.1	8	47.0
Master’s degree	2	28.6	6	35.3
Doctorate degree	0	0.0	1	5.9
Annual household income				
$50,000 to $74,999	0	0.0	1	5.9
$75,000 to $99,999	0	0.0	1	5.9
$100,000 to $149,999	2	28.6	2	11.8
$150,000 to $199,999	0	0.0	3	17.6
$200,000 to $249,999	1	14.2	4	23.5
Over $250,000	2	28.6	4	23.5
Do not wish to answer	2	28.6	2	11.8
Job location				
Office	6	85.7	14	82.4
Laboratory	1	14.3	0	0.0
Field-based office	0	0.0	3	17.6
Work demand				
Easy	0	0.0	1	5.9
Moderate	1	14.3	5	29.4
Demanding	2	28.6	4	23.5
Very demanding	4	57.1	7	41.2
Employment status				
Part-time active	0	0.0	1	5.9
Full-time active	7	100.0	16	94.1
Primary cancer site				
Breast cancer	4	57.1	6	35.1
Gynecological	0	0.0	2	11.8
Kidney	0	0.0	2	11.8
Lymph nodes	0	0.0	2	11.8
Thyroid	0	0.0	2	11.8
Cancer stage				
0	0	0	4	23.5
1	3	42.8	3	17.7
2	1	14.3	5	29.4
3	2	28.6	5	29.4
4	1	14.3	0	0
Time since diagnosis				
Less than 6 months	1	14.3	2	11.8
6 to 12 months	0	0.0	1	5.9
1 to 2 years	3	42.8	1	5.9
2 to 5 years	2	28.6	3	17.6
5 to 10 years	0	0.0	6	35.3
Over 10 years	1	14.3	4	23.5
Type of treatment				
Chemotherapy (only)	0	0	2	11.8
Radiation (only)	0	0	1	5.9
Surgery (only)	1	14.4	4	23.5
2 or more treatment types	3	42.8	3	17.6
3 or more treatment types	3	42.8	7	41.2
Current treatment phase				
Active treatment	4	57.2	2	11.8
No treatment	3	42.8	15	88.2

### Survey outcomes

Attitudinal self-efficacy, emotional support, and informational support (measured by CASE-cancer and PROMIS-Social Support) were not statistically different between program users and non-users (Table 2) [8, 11, 12]. Program users rated their relationship with the Nurse Navigator (measured by PSN-I) highly, with a mean score of 38.86 out of a possible score of 45 (Table 2) [8, 11, 12]. When outcomes for all participants (*N* = 24) were grouped according to demographic variables, the largest numerical difference was for emotional support according to marital status: the mean score was nearly 5 points higher for participants who were married (34.2) versus not (29.31) (Table 3) [8, 11].

**Table 2:** Study outcomes: self-efficacy, social support, and patient satisfaction

	Program users (*N* = 7)	Non-users (*N* = 17)	*p* value (95% confidence interval)
CASE-cancer*			
Mean (SD)	38.43 (5.77)	41.65 (4.65)	0.16 (–7.86, 1.42)
Median	36	42	
PROMIS-Social Support: Emotional Support^†^			
Mean (SD)	31.43 (5.50)	32.94 (5.51)	0.55 (– 6.64, 3.61)
Median	30	35	
PROMIS-Social Support: Informational Support^†^			
Mean (SD)	30.71 (5.47)	30.35 (5.06)	0.88 (– 4.46, 5.18)
Median	30	31	
PSN-I^‡^			
Mean (SD)	38.86 (6.18)		
Median	41		

*CASE-cancer* Communication and Attitudinal Self-Efficacy for Cancer, *PROMIS* Patient-Reported Outcomes Measurement Information System, *PSN-I* Patient Satisfaction with Interpersonal Relationship with Navigator, *SD* standard deviation

*Adapted from [8]. Possible range of scores is 12 to 48

^†^Adapted from [11]. Possible range of scores for each domain is 8 to 40

^‡^Adapted from [12]. Possible range of scores is 9 to 45

**Table 3:** Study outcomes by demographic variables

Demographic variable	Mean scores by demographic category Ensemble users and non-users (Total *N* = 24)		
	Self-efficacy*	Emotional support^†^	Informational support^†^
Gender			
Male	40.00	34.00	30.85
Female	41.00	31.88	30.29
Marital status			
Married	40.53	34.20	30.80
Not married	42.21	29.31	29.85
Level of education			
Associate’s/Baccalaureate	39.67	30.33	28.96
Master’s/Doctorate	43.44	28.88	27.06
Cancer stage			
0 to 1	40.38	32.54	31.33
2 to 4	39.37	31.54	30.08
Time since diagnosis			
< 1 year	40.67	29.33	29.50
> 1 year	40.17	32.70	30.54
Treatment phase			
In treatment	39.67	34.83	32.50
Not in treatment	41.06	31.72	29.78

*Adapted from [8]. Possible range of scores is 12 to 48

^†^Adapted from [11]. Possible range of scores for each domain is 8 to 40

### Responses to open-ended questions

**Fig. 1:**
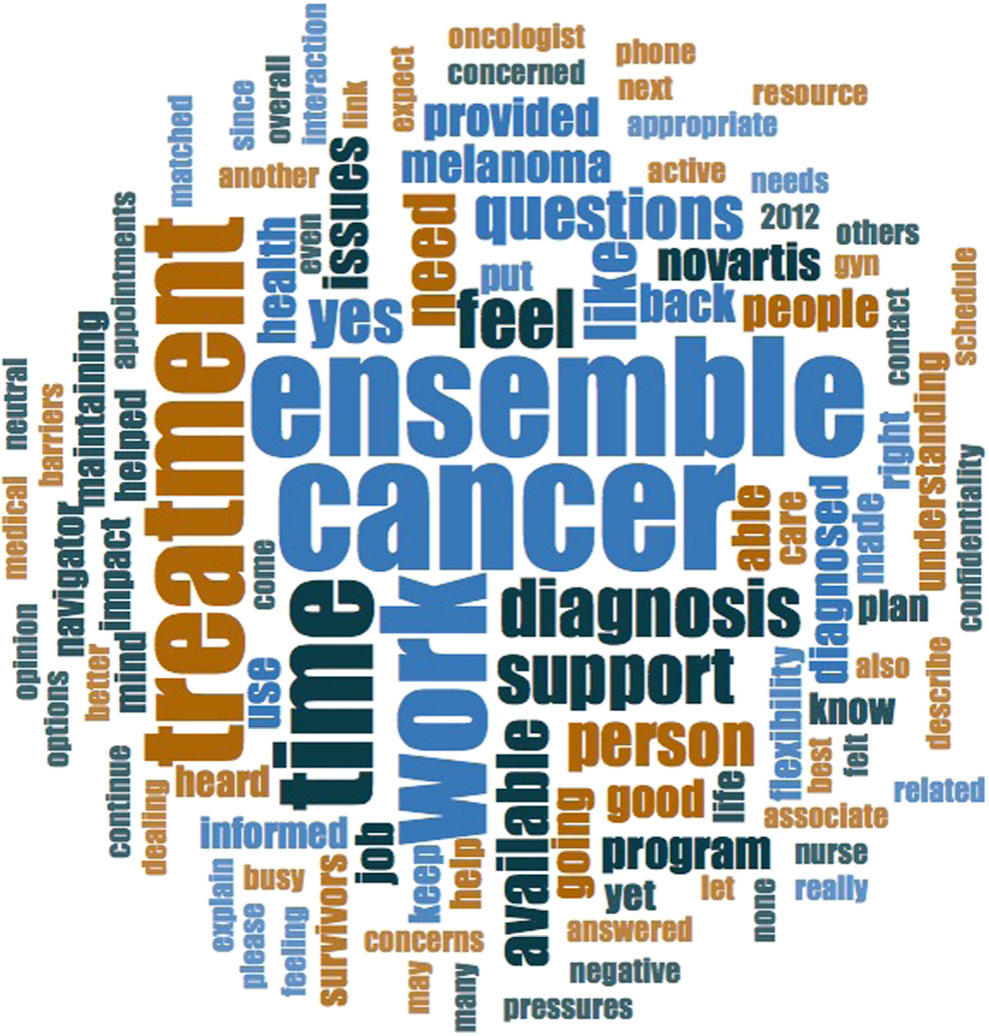
A word cloud illustrating responses to open-ended questions that were generated from the responses to the open-ended questions using qualitative software analysis program NVivo (Version 11)

The five most frequently cited words in the open-ended responses (Fig. 1) were cancer, Ensemble, work, treatment, and time. Notably, words regarding the program’s key elements were noted infrequently, including navigator (3 times), nurse (2 times), and the specific name of a Nurse Navigator (1 time). Participants who discussed the program support often referred to the administrator of support as “Ensemble.”

Participants reported that health issues compounded the stress from high work demands. Several non-users stated that they did not use the Ensemble program because they were too busy with work. Protection of privacy was the most cited barrier to using the program and was accompanied by concerns of cancer-related stigma. However, program users expressed gratitude that the Ensemble program was available and felt it was beneficial because “having a relatively anonymous, non-judgmental person to interact with” provided “someone to support me in my professional life.” One user stated: “My worries and concerns were listened to without judgment, which was a big relief.”

Participants reported returning to normalcy as an important aspect of returning to work. Accordingly, some participants believed that a cancer support program should be separate from the work setting. Maintaining optimal health was a challenge to individuals attempting to sustain a demanding work schedule, with stress and anxiety, fatigue, and maintaining a healthful lifestyle among the top-stated concerns. Stress in particular was identified as affecting overall well-being. Ensemble users reported that the program provided helpful information, emotional support, and guidance to maintain optimal health, with emotional support resulting in the greatest impact. Most study participants addressed the importance of social support at work. In addition to work flexibility, cancer survivors stated that the understanding and ability of “supervisors who are willing to work with me” were very important. “Feeling supported by my boss…matters.”

## Discussion

Finding solutions that facilitate return to work for cancer survivors is imperative for their long-term well-being. A rigorous systematic review and meta-synthesis of qualitative studies indicate that work reinforces self-identity through increased self-esteem, financial security, and social support, and by demonstrating a person’s abilities, talents, and health [3]. Ensemble, an innovative workplace program based on a navigation model using nurses, aims to provide emotional and informational support along with health coaching to improve self-efficacy and better equip participants to cope with the complex demands of balancing work and a cancer diagnosis.

A key limitation in this study was the limited sample size because of low usage rates for the Ensemble program. In addition, few non-users participated in the study. As a result, quantitative data lacked sufficient power to form conclusions regarding self-efficacy, social support, patient satisfaction, or their relationships to demographic variables. Participation may have been influenced by immediate need (a larger proportion of program users [4/7] were in active treatment compared with non-users [2/17]), privacy concerns, stigma, and high work demands. Current participation remains low (~ 4% of cancer survivors); along with privacy concerns, this may be attributed to the fact that most individuals with cancer in the USA do not use formal support programs [14, 15]. In an attempt to alleviate privacy concerns and elevate the level of expertise involved, the Ensemble program is currently being administered via Johns Hopkins Medicine. It is also possible that the need for a program like Ensemble might be greater in work settings with less access to supportive care overall; however, additional research is needed to determine the potential drivers for use of such interventions.

Although the information and resources of the Ensemble program are specific to the organization of the author, the program framework of using a Nurse Navigator for individualized care and a company website with specific resources for cancer survivors can easily be adapted to other organizations. One aspect of the program that may not be as readily adapted to other organizations is the use of internal medical experts as volunteers to provide guidance and support to their fellow employees. Instead, other organizations might need external experts or resources to provide the same guidance. For example, Johns Hopkins Medicine is currently providing a nurse navigational program as a benefit to its employees with cancer and has made this available to other employers as an online resource (https://www.workstride.org/). Instead of medical experts as one-on-one volunteers, the Johns Hopkins program includes a comprehensive website created and regularly maintained by experts in the fields such as breast cancer oncologists and nurses.

Investigational work support interventions for cancer survivors in other countries include occupational counseling and rehabilitation [16–19]. For example, a prospective study in the Netherlands reported high return-to-work rates for cancer survivors (83% at 18 months) and reduced fatigue with a multidisciplinary intervention using physical exercise and occupational counseling [16]. A Belgian study proposed a return-to-work intervention for breast cancer patients using occupational therapists to bridge healthcare and the workplace [20]. A recent review of the literature concluded that global efforts are needed to implement routine cancer rehabilitation and survivorship care [21].

An aspect of cancer survivorship that we were not able to investigate in this study that results in significant work impairment is lymphedema, a potential complication of cancer and its treatment (especially surgery) [22]. Participants in a qualitative study on the experiences of Australian cancer survivors with lymphedema indicated that maintaining work was important for their identity and emphasized their need for privacy regarding their diagnosis [23]. The three most common factors cited by a group of Austrian experts for improving staying at or returning to work with lymphedema were early rehabilitation, self-management/coping strategies/patient education, and the goodwill/cooperation of the employer [24].

The goal of this report is to provide a perspective on the perceptions of individuals with cancer who continue to work, to aid in the development of effective work navigational programs, and to inform future research into workplace intervention programs for cancer survivors. Mean self-efficacy and social support scores among all participants were in the middle range of possible scores and open-ended responses confirmed the need for social support at work, highlighting the importance of improving these parameters among cancer survivors who continue to work. Recent studies demonstrate that a robust sense of self-efficacy provides strong emotional health and ability to adjust to work after cancer [25, 26]. Likewise, social support at work is an important factor in the cancer and work experience, as demonstrated by the Cancer Survivorship and Work Model [13]. Enhancing a sense of self-efficacy is a valuable target for workplace intervention programs, and social support should remain a key objective of program improvement and research outcomes.

Measuring the program users’ satisfaction with the interpersonal relationship was important because the Nurse Navigators were the central feature of the navigation process. In addition to high scores on the PSN-I, which measures satisfaction in the relational aspect of navigation [12], in their open-ended responses, program users expressed that the navigators listened, acted, and assisted in the individuals’ ability to work. This high level of satisfaction among users supports the application of a navigation program across work settings, further improving and extending the care and relational alliance provided by Nurse Navigators.

Common themes among open-ended responses centered around work demands, privacy, integration of life and work, and program improvement and may facilitate program development and improvement. Recommendations include continuing to use the framework of navigation for the program and expanding the program’s services to include the full trajectory of cancer, from more intensive support services for the newly diagnosed to support for long-term health. Nurse Navigators should continue to provide emotional and informational support, self-efficacy coaching, advocacy, referrals to resources, and solutions to instrumental barriers for cancer survivors who continue to work. Participants also recommended that employers provide flexibility to accommodate appointments, treatments, and fluctuations in work ability. Indeed, employers need education on the importance of flexibility and supporting employees for optimal outcomes for both the cancer survivor and the employer. In addition to the cancer survivor-focused interventions, the Ensemble program also provides information and support to managers who have employees with cancer so they can understand how best to support them.

Overall, observations from this study provide information for future intervention, patient-reported outcomes research, and innovation to support the growing number of cancer survivors returning to work.

## Electronic supplementary material

Online Resource 1 Summary of study design (PDF 106 kb)

Online Resource 2 Demographic Questionnaire (PDF 120 kb)

Online Resource 3 Open-ended questions (PDF 74 kb)

Online Resource 4 Sampling and flow of study participation (PDF 123 kb)

Online Resource 5 The Cancer Survivorship and Work Model (PDF 94 kb)

## Data Availability

All data generated or analyzed during this study are included in this published article and its supplementary information files.

## References

[1] Siegel RL, Miller KD, Jemal A (2018). Cancer statistics, 2018. CA Cancer J Clin.

[2] Amir Z, Brocky J (2009). Cancer survivorship and employment: epidemiology. Occup Med (Lond).

[3] Wells M, Williams B, Firnigl D, Lang H, Coyle J, Kroll T, MacGillivray S (2013). Supporting ‘work-related goals’ rather than ‘return to work’ after cancer? A systematic review and meta-synthesis of 25 qualitative studies. Psychooncology.

[4] Mehnert A (2011). Employment and work-related issues in cancer survivors. Crit Rev Oncol Hematol.

[5] Bilodeau K, Tremblay D, Durand MJ (2017). Exploration of return-to-work interventions for breast cancer patients: a scoping review. Support Care Cancer.

[6] Wells Kristen J., Battaglia Tracy A., Dudley Donald J., Garcia Roland, Greene Amanda, Calhoun Elizabeth, Mandelblatt Jeanne S., Paskett Electra D., Raich Peter C. (2008). Patient navigation: State of the art or is it science?. Cancer.

[7] McBrien KA, Ivers N, Barnieh L, Bailey JJ, Lorenzetti DL, Nicholas D, Tonelli M, Hemmelgarn B, Lewanczuk R, Edwards A, Braun T, Manns B (2018). Patient navigators for people with chronic disease: a systematic review. PLoS One.

[8] Wolf MS, Chang CH, Davis T, Makoul G (2005). Development and validation of the Communication and Attitudinal Self-Efficacy scale for cancer (CASE-cancer). Patient Educ Couns.

[9] Hahn Elizabeth A., DeVellis Robert F., Bode Rita K., Garcia Sofia F., Castel Liana D., Eisen Susan V., Bosworth Hayden B., Heinemann Allen W., Rothrock Nan, Cella David (2010). Measuring social health in the patient-reported outcomes measurement information system (PROMIS): item bank development and testing. Quality of Life Research.

[10] Hahn EA, DeWalt DA, Bode RK, Garcia SF, DeVellis RF, Correia H, Cella D, PROMIS Cooperative Group (2014) New English and Spanish social health measures will facilitate evaluating health determinants. Health Psychol 33:490–499 10.1037/hea0000055 PMC4159098 24447188

[11] Patient Reported Outcomes Measurement Information System (PROMIS) Domain framework-social health (2014) Available at: http://nihpromis.org/measures/domainframework3 Accessed 28 Feb 2018

[12] Jean-Pierre P, Fiscella K, Winters PC, Post D, Wells KJ, McKoy JM, Battaglia T, Simon MA, Kilbourn K, Patient Navigation Research Program Group(2012) Psychometric development and reliability analysis of a patient satisfaction with interpersonal relationship with navigator measure: a multi-site patient navigation research program study. Psychooncology 21:986–992 10.1002/pon.2002 PMC3640800 21681995

[13] Mehnert A, de Boer A, Feuerstein M (2013). Employment challenges for cancer survivors. Cancer.

[14] Kumar P, Casarett D, Corcoran A, Desai K, Li Q, Chen J, Langer C, Mao JJ (2012). Utilization of supportive and palliative care services among oncology outpatients at one academic cancer center: determinants of use and barriers to access. J Palliat Med.

[15] Sherman AC, Pennington J, Simonton S, Latif U, Arent L, Farley H (2008). Determinants of participation in cancer support groups: the role of health beliefs. Int J Behav Med.

[16] Leensen MCJ, Groeneveld IF, Heide IV, Rejda T, van Veldhoven PLJ, Berkel SV, Snoek A, Harten WV, Frings-Dresen MHW, de Boer A (2017). Return to work of cancer patients after a multidisciplinary intervention including occupational counselling and physical exercise in cancer patients: a prospective study in the Netherlands. BMJ Open.

[17] Tamminga SJ, Verbeek JH, Bos MM, Fons G, Kitzen JJ, Plaisier PW, Frings-Dresen MH, de Boer AG (2013). Effectiveness of a hospital-based work support intervention for female cancer patients - a multi-centre randomised controlled trial. PLoS One.

[18] Wolvers MDJ, Leensen MCJ, Groeneveld IF, Frings-Dresen MHW, De Boer A (2018). Predictors for earlier return to work of cancer patients. J Cancer Surviv.

[19] Wolvers M. D. J., Leensen M. C. J., Groeneveld I. F., Frings-Dresen M. H. W., De Boer A. G. E. M. (2018). Longitudinal Associations Between Fatigue and Perceived Work Ability in Cancer Survivors. Journal of Occupational Rehabilitation.

[20] Desiron HA, Crutzen R, Godderis L, Van Hoof E, de Rijk A (2016). Bridging health care and the workplace: formulation of a return-to-work intervention for breast cancer patients using an intervention mapping approach. J Occup Rehabil.

[21] Smith SR, Zheng JY, Silver J, Haig AJ, Cheville A (2018) Cancer rehabilitation as an essential component of quality care and survivorship from an international perspective. Disabil Rehabil. 10.1080/09638288.2018.1514662 10.1080/09638288.2018.1514662 30574818

[22] Boyages J, Kalfa S, Xu Y, Koelmeyer L, Mackie H, Viveros H, Taksa L, Gollan P (2016). Worse and worse off: the impact of lymphedema on work and career after breast cancer. Springerplus.

[23] Kalfa Senia, Koelmeyer Louise, Taksa Lucy, Winch Caleb, Viveros Hector, Gollan Paul J., Mackie Helen, Boyages John (2018). Work experiences of Australian cancer survivors with lymphoedema: A qualitative study. Health & Social Care in the Community.

[24] Neubauer M, Schoberwalter D, Cenik F, Keilani M, Crevenna R (2017). Lymphedema and employability - review and results of a survey of Austrian experts. Wien Klin Wochenschr.

[25] Rottmann N, Dalton SO, Christensen J, Frederiksen K, Johansen C (2010). Self-efficacy, adjustment style and well-being in breast cancer patients: a longitudinal study. Qual Life Res.

[26] Philip EJ, Merluzzi TV, Zhang Z, Heitzmann CA (2013). Depression and cancer survivorship: importance of coping self-efficacy in post-treatment survivors. Psychooncology.

