# Supplementary material for: Patient-reported outcomes from a workplace intervention program for cancer survivors highlight ongoing needs to support continuation of work
Source: Support Care Cancer. 2019 Jul 8;27(11):4377–84. doi: 10.1007/s00520-019-04964-1 (PMC6803589; doi:10.1007/s00520-019-04964-1)
Supplement: Supplementary file 1 — Summary of study design (PDF 106 kb) [file 520_2019_4964_MOESM1_ESM.pdf]

Dawn Aubel
Novartis Oncology, East Hanover, NJ
dawn.aubel@novartis.com

Online Resource 1 Summary of study design

Group	Survey
Ensemble program users	CASE-cancer*
	PROMIS-Social Support†
	PSN-I‡
	Demographic questionnaire
	Open-Ended questions
Ensemble program non-users	CASE-cancer*
	PROMIS-Social Support†
	Demographic questionnaire
	Open-ended questions

CASE-cancer Communication and Attitudinal Self-Efficacy for Cancer, *PROMIS* Patient-Reported Outcomes Measurement Information System, *PSN-I* Patient Satisfaction with Interpersonal Relationship with Navigator, *SD* standard deviation
*Adapted from Wolf MS, Chang CH, Davis T, Makoul G (2005) Development and validation of the Communication and Attitudinal Self-Efficacy scale for cancer (CASE-cancer). Patient Educ Couns 57(3):333-341
†Adapted from Patient Reported Outcomes Measurement Information System (PROMIS). Domain framework- Social health (2014) http://nihpromis.org/measures/domainframework3
‡Adapted from Jean-Pierre P, Fiscella K, Winters PC, Post D, Wells KJ, McKoy JM, Battaglia T, Simon MA, Kilbourn K; Patient Navigation Research Program Group (2012) Psychometric development and reliability analysis of a patient satisfaction with interpersonal relationship with navigator measure: a multi-site patient navigation research program study. Psychooncology 21(9):986-992
