# Supplementary material for: Patient-reported outcomes from a workplace intervention program for cancer survivors highlight ongoing needs to support continuation of work
Source: Support Care Cancer. 2019 Jul 8;27(11):4377–84. doi: 10.1007/s00520-019-04964-1 (PMC6803589; doi:10.1007/s00520-019-04964-1)
Supplement: Supplementary file 2 — Demographic Questionnaire (PDF 120 kb) [file 520_2019_4964_MOESM2_ESM.pdf]

Online Resource 2

Dawn Aubel
Novartis Oncology, East Hanover, NJ
dawn.aubel@novartis.com

Demographic Questionnaire

Some of the following data may be information you wish not to reveal. This is acceptable, however when more information is provided the depth of understanding improves. Please choose one best response for each question that you choose to answer:

1. What is your age?
   a. Under 25
   b. 25 to 29
   c. 30 to 34
   d. 35 to 39
   e. 40 to 44
   f. 45 to 49
   g. 50 to 54
   h. 55 to 59
   i. 60 to 64
   j. Over 65
   k. Do not wish to answer

2. What is your gender?
   a. Male
   b. Female
   c. Do not wish to answer

3. What is your primary language?
   a. English
   b. Spanish
   c. Mandarin
   d. Indian
   e. Arabic
   f. French
   g. Do not wish to answer

4. What is your ethnicity?
   a. African American/Black
   b. Asian
   c. Asian/Pacific Islander
   d. Caucasian/White
   e. Hispanic
   f. Native American
   g. Other
   h. Do not wish to answer

5. What is your marital status?
   a. Single/never married
   b. Married/living as married
   c. Divorced/separated
   d. Widowed
   e. Do not wish to answer

6. What is the highest level of education that you have completed?
   a. Less than high school
   b. High school diploma
   c. Some college/vocational after high school
   d. Associate degree
   e. Baccalaureate degree
   f. Master's degree
   g. Doctorate degree
   h. Do not wish to answer

7. What is your annual household income?
   a. Less than $25,000
   b. $25,000 to $49,999
   c. $50,000 to $74,999
   d. $75,000 to $99,999
   e. $100,000 to $149,999
   f. $150,000 to $199,999
   g. $200,000 to $249,999
   h. Over $250,000
   i. Do not wish to answer

8. What is your primary cancer site?
   a. Blood/Hematologic
   b. Breast
   c. Gastrointestinal
   d. Gynecological
   e. Head and neck
   f. Kidney
   g. Lung
   h. Lymph nodes
   i. Melanoma
   j. Mixed
   k. Prostate
   l. Testicular
   m. Thyroid
   n. Urinary
   o. Other
   p. Unknown

9. What is your cancer stage?
   a. 0
   b. 1
   c. 2
   d. 3
   e. 4
   f. Unknown

10. What is your time since diagnosis?
    a. Less than 6 months
    b. 6 to 12 months
    c. 1 to 2 years
    d. 2 to 5 years
    e. 5 to 10 years
    f. Over 10 years

11. What type of treatment are you in or have you had?
    a. Chemotherapy (intravenous)
    b. Chemotherapy (by mouth)
    c. Hormonal
    d. Radiation
    e. Surgery
    f. Combination

12. What is your treatment phase at this time?
    a. Active treatment
    b. No treatment

13. Where is your job based?
    a. Office
    b. Laboratory
    c. Manufacturing
    d. Field-based office
    e. Other

14. What is the work demand?
    a. Extremely easy
    b. Easy
    c. Moderate
    d. Demanding
    e. Very demanding

15. What is your employment status?
    a. Part-time active
    b. Part-time inactive
    c. Full-time active
    d. Full-time inactive
    e. Retired
