# Supplementary material for: Patient-reported outcomes from a workplace intervention program for cancer survivors highlight ongoing needs to support continuation of work
Source: Support Care Cancer. 2019 Jul 8;27(11):4377–84. doi: 10.1007/s00520-019-04964-1 (PMC6803589; doi:10.1007/s00520-019-04964-1)
Supplement: Supplementary file 3 — Open-ended questions (PDF 74 kb) [file 520_2019_4964_MOESM3_ESM.pdf]

Dawn Aubel
Novartis Oncology, East Hanover, NJ
dawn.aubel@novartis.com

Online Resource 3 Open-ended questions

Ensemble program users

1. What aspects of Ensemble were the most beneficial for you?
2. Describe how Ensemble impacted your work and/or overall health.
3. What specific suggestions do you have for the program improvement?

Ensemble program non-users

1. Please describe your most important needs related to working after a cancer diagnosis.
2. If a workplace program for cancer survivors was offered by your employer, what would be the most important services provided?
3. In your opinion, what would be the barriers to participation in a workplace program for cancer survivors?
