# Supplementary material for: Patient-reported outcomes from a workplace intervention program for cancer survivors highlight ongoing needs to support continuation of work
Source: Support Care Cancer. 2019 Jul 8;27(11):4377–84. doi: 10.1007/s00520-019-04964-1 (PMC6803589; doi:10.1007/s00520-019-04964-1)
Supplement: Supplementary file 5 — The Cancer Survivorship and Work Model (PDF 94 kb) [file 520_2019_4964_MOESM5_ESM.pdf]

Dawn Aubel
Novartis Oncology, East Hanover, NJ
dawn.aubel@novartis.com

Online Resource 5 The Cancer Survivorship and Work Model

Policies, procedures, and economic factors that may affect employment and return to work*

Factors Related to the Work Environment	Factors Related to the Cancer Survivor	Interventions and Rehabilitation Programs Promoting Return to Work and Employment	Outcomes
Work conditions<br>Work demands<br>Employer accommodation<br>Work climate<br>Flexibility<br>Work gratification	**Individual and Interpersonal Factors**<br>• Sociodemographics<br>• Socioeconomic status<br>• Educational and Professional training<br>• Life stage<br>• Personality<br>• Coping Strategies<br>• Problem-solving skills<br>• Motivation<br>• Meaning of work<br>• Social supports<br>**Short-term, long-term, and late effects of cancer and treatments**<br>• Health status/comorbidity<br>• Continuity of care<br>• Quality of life<br>• Functional impairments<br>• Symptom burden<br>• Emotional and social well being<br>• Change in identity and role functioning<br>• Social reintegration	• Improvement of physical fitness and psychosocial functioning<br>• Assessment and evaluation of work-related skills and work demands<br>• Skills training (eg, job application training)<br>• Occupational counseling and education<br>• Employer education and counseling	• Employment/Return to work<br>• Work ability<br>• Work performance<br>• Job opportunities<br>• Income<br>• Work satisfaction<br>• Job promotion and training<br>• Sustainability

*Based on Mehnert A, de Boer A, Feuerstein M (2013) Employment challenges for cancer survivors. Cancer 119(Suppl 11):2151-2159
